# Sensorimotor Recalibration Depends on Attribution of Sensory Prediction Errors to Internal Causes

**DOI:** 10.1371/journal.pone.0054925

**Published:** 2013-01-24

**Authors:** Carlo Wilke, Matthis Synofzik, Axel Lindner

**Affiliations:** 1 Department of Cognitive Neurology, Hertie Institute for Clinical Brain Research, University of Tübingen, Tübingen, Germany; 2 Department of Neurodegeneration, Hertie Institute for Clinical Brain Research, University of Tübingen, Tübingen, Germany; 3 German Centre for Neurodegenerative Diseases, University of Tübingen, Tübingen, Germany; ICREA-University of Barcelona, Spain

## Abstract

Sensorimotor learning critically depends on error signals. Learning usually tries to minimise these error signals to guarantee optimal performance. Errors can, however, have both *internal* causes, resulting from one’s sensorimotor system, and *external* causes, resulting from external disturbances. Does learning take into account the *perceived cause* of error information? Here, we investigated the recalibration of internal predictions about the sensory consequences of one’s actions. Since these predictions underlie the distinction of self- and externally produced sensory events, we assumed them to be recalibrated only by prediction errors attributed to internal causes. When subjects were confronted with experimentally induced visual prediction errors about their pointing movements in virtual reality, they recalibrated the predicted visual consequences of their movements. Recalibration was not proportional to the externally generated prediction error, but correlated with the error component which subjects attributed to *internal* causes. We also revealed adaptation in subjects’ motor performance which reflected their recalibrated sensory predictions. Thus, causal *attribution* of error information is essential for sensorimotor learning.

## Introduction

**Figure 1:**
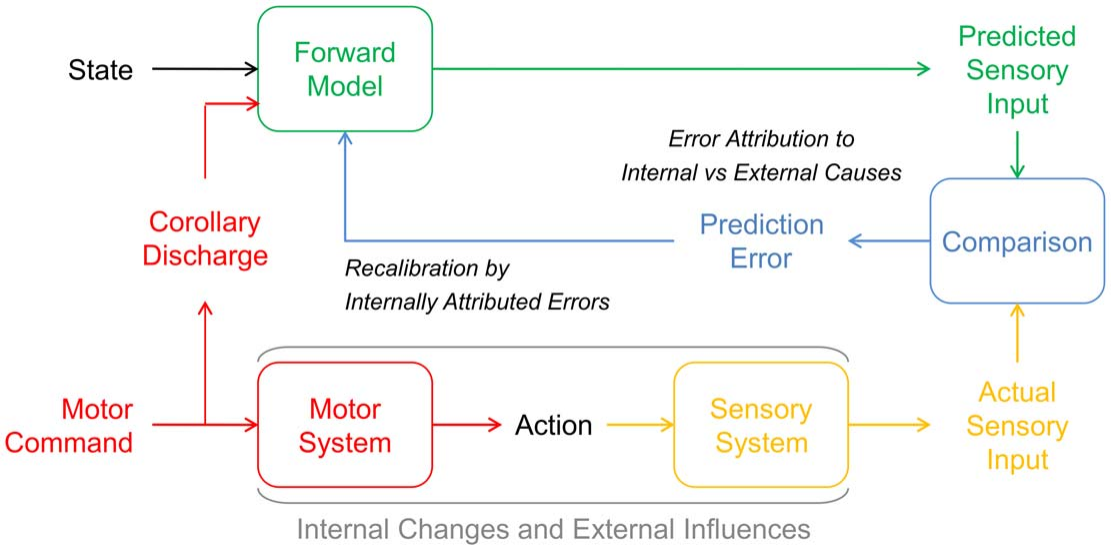
Recalibration of internal sensory predictions. Sensory afference can result both from external events (exafference) and, as this figure illustrates, from our own actions (reafference) [1]. According to the comparator model, the nervous system establishes the cause of the sensory afference by comparing the *actual sensory input* with the *predicted sensory input* [2], [3]. To this end, the sensorimotor system predicts the sensory input which will result from one’s actions on the basis of internal action-related information, such as *corollary discharge* [5] of the *motor command*. This prediction is computed by a *forward model* [6], [7] which additionally takes into account the current *state* of the *motor system* and the *sensory system* [34], [64]. The nervous system then makes a *comparison* between the *actual sensory input* and the *predicted sensory input*. In case of a match, the sensory afference should be interpreted as internally caused. Otherwise, in case of a mismatch, the difference between the actual and the predicted input should be interpreted as externally caused. This difference between the actual and the internally predicted sensory consequences of one’s actions constitutes a *prediction error*. However, such errors arise not only from *external influences*. Prediction errors can also result from *internal changes*, i.e. changes within the sensorimotor system such as growth, fatigue or disease. Thus, one’s internal sensory predictions need continuous *recalibration*. As previous research suggests [16], [43], [65], this recalibration should compensate only for those prediction errors which result from internal causes. However, internally and externally caused prediction errors do not differ per se. Addressing this issue, we here demonstrate that the recalibration of internal sensory predictions by prediction errors depends on the attribution of the prediction error to internal causes. Figure adapted from Wolpert and Miall, 1996 [66].

Sensory information results from both external events and our own actions [1]. To distinguish between *externally* and *internally caused* sensory information [2], [3], the nervous system predicts the sensory consequences of one’s actions on the basis of internal action-related information, such as efference copies [4] or corollary discharge [5] of motor commands. These *internal sensory predictions* are likely issued by forward models [6], [7] which take into account the current states of the motor and the sensory system. By comparing the actual and the predicted sensory afference, the nervous system can infer the cause of the afference [2], [3]. In case of a match, the afference should be interpreted as internally caused. Otherwise, the difference between the actual and the predicted afference – i.e. the *prediction error* – should be interpreted as the result of an external event (compare Figure 1).

However, as the properties of the motor system continuously change (e.g. due to growth, fatigue or disease), one and the same motor command will have various sensory consequences [8]. Such changes will likewise produce prediction errors – unlike in the first case, however, these errors result not from external events, but from internal causes. In order to maintain a reliable distinction between externally and internally caused sensory afference, internal sensory predictions therefore need continuous *recalibration* [8], [9], [10], [11], [12] to compensate for internally caused prediction errors. Such recalibration constitutes a fundamental problem since internally and externally caused prediction errors do not differ per se.

Given this uncertainty about the cause of prediction errors, we here asked whether the recalibration of internal sensory predictions depends on the *attribution* of prediction errors to internal causes (i.e. causes within the sensorimotor system). We therefore designed a sensorimotor recalibration paradigm and estimated the component of the prediction error which subjects attributed to internal causes. Our findings demonstrate that this *internally attributed component* determines the recalibration of internal sensory predictions.

## Results

### Causal Attribution of Sensory Prediction Errors

**Figure 2:**
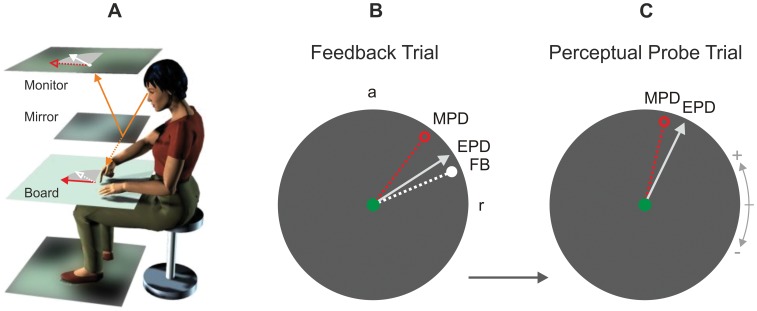
Experimental design. *(A) Setup.* Subjects viewed the virtual image of their finger (white disc) on the feedback monitor via a mirror (solid orange arrow) while performing horizontal pointing movements. For geometric reasons, the virtual image appeared in the same plane as subjects’ finger movements (dotted orange arrow). Visual feedback could be either veridical, i.e. in spatiotemporal correspondence with subjects’ fingertip, or manipulated online by rotation around the starting point of the movements (solid red arrow: actual movement vector, solid white arrow: rotated visual feedback vector, dotted arrows correspond to projections of these vectors into the monitor or the movement plane, respectively). *(B) and (C) Procedure.* Feedback trials (B) and perceptual probe trials (C) followed on each other alternately. In both conditions, subjects were instructed to freely choose various motor pointing directions between the subjective directions of *right* (r = 0°) and *anterior* (a = 90°). In feedback trials (B), visual feedback (FB, dotted white line) about the pointing movement (MPD, dotted red line) was provided in real time. Feedback could be rotated around the starting point (green disc) of the movements by various angles, either in a clockwise (as in this example) or in a counterclockwise manner. When having completed a movement, subjects visually estimated the direction of their movement (referred to as the estimated pointing direction, EPD, solid grey arrow) by placing a trackball-guided cursor in the respective direction. The perceived pointing direction, defined as the difference PPD = EPD – MPD, allowed to us to estimate the component of the feedback manipulation – and thus of the visual prediction error – which subjects attributed to internal causes. In perceptual probe trials (C), subjects did not receive any visual feedback about their pointing movement (MPD). Consequently, they needed to rely entirely on internal action-related information when estimating their pointing direction (EPD). By analysing subjects’ perceived pointing direction (PPD = EPD – MPD) in perceptual probe trials as a function of the visual manipulation applied in the preceding feedback trial, we assessed how subjects’ internal sensory predictions recalibrated in response to visual prediction errors (see Figure 1 for background information).

We tested our hypothesis in a virtual-reality setup (Figure 2A, [8]) in which subjects (n = 11) performed horizontal pointing movements in freely chosen directions. To generate visual prediction errors and to manipulate the component of these errors which subjects attributed to internal causes, we used trials with online visual feedback – referred to as *feedback trials* (Figure 2B). Here, subjects were provided with a visual marker which moved in an experimentally controlled relation to the tip of their right index finger. This visual feedback (FB) was either veridical, i.e. in spatiotemporal correspondence with the fingertip, or rotated relative to the actual movement by various angles. For each trial, the manipulation angle was randomly drawn from a discrete uniform distribution over the values 5°, 10°, 20°, 40° (counterclockwise rotation), −5°, −10°, −20°, −40° (clockwise rotation) and 0° (veridical feedback). Subjects were informed in advance that the visual feedback would be either veridical or rotated relative to their movements by various angles. Given the unpredictability and the distribution of the visual manipulation, subjects’ internal prediction of the visual pointing direction should on average have corresponded to the direction of the actually performed movement. Accordingly, any manipulated feedback should have deviated from this internal prediction, which means that *feedback manipulations* should constitute visual prediction errors which are externally caused.

**Figure 3:**
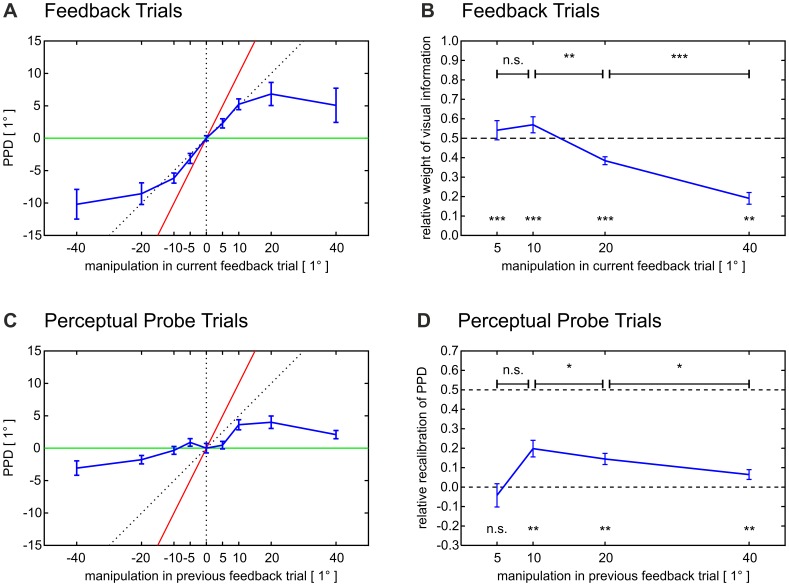
Error attribution and recalibration of internal sensory predictions. *(A) Perceived pointing direction in feedback trials.* Subjects’ perceived pointing direction (PPD, blue curve), which is here plotted versus the manipulation applied to the visual feedback, allowed us to estimate the component of the feedback manipulation – and thus of the visual prediction error – which subjects attributed to internal causes. The PPD strongly reflected the size of the feedback manipulation (red line) if these manipulations were small. If manipulations were large, vice versa, subjects’ estimated pointing direction rather resembled the motor pointing direction (green line). By varying the size of the feedback manipulations, we thus gradually manipulated the error component which subjects attributed to internal causes. *(B) Relative weight of visual information.* We captured the internally attributed share of the prediction error by the relative weight which visual feedback obtained in subjects’ PPD. The relative weight of visual information was defined as the quotient of subjects’ offset-corrected PPD (compare A) and the manipulation applied to the visual feedback. Relative visual weights of 1 and 0 would indicate that a subject attributed the prediction error either entirely internally or, respectively, entirely externally. The relative visual weight was significant for all amounts of manipulation (one-sample one-tailed t-tests), but also quantitatively modulated by the absolute amount of manipulation: increasing error sizes resulted in decreasing shares of the internally attributed error component (paired one-tailed t-tests). *(C) Perceived pointing direction in perceptual probe trials.* The recalibration of subjects’ internal sensory predictions was reflected by the PPD in perceptual probe trials (blue curve), which is here plotted versus the feedback manipulation applied in the immediately preceding feedback trial. Recalibration was not proportional to the preceding prediction error, i.e. the preceding feedback manipulation, but instead resembled the error component which subjects had attributed internally, i.e. their PPD in the preceding feedback trial (compare A). By way of illustration, the green line shows the assumption that prediction errors would not induce any recalibration. Likewise, the red line corresponds to the assumption that subjects adjusted the perceived pointing direction to the entire amount of the preceding feedback manipulation. *(D) Relative recalibration of internal sensory predictions.* We defined the relative recalibration to compare the recalibration induced by prediction errors of variable size. The relative recalibration was the quotient of the offset-corrected PPD in a given perceptual probe trial (compare C) and the manipulation applied in the preceding feedback trial. The relative recalibration was significant for amounts of manipulation as large as 10°, 20° and 40° (one-sample one-tailed t-tests), which means that manipulations induced recalibration if exceeding a minimum threshold. Moreover, the amount of manipulation modulated the relative recalibration quantitatively: increasing amounts of manipulation resulted in decreasing values of relative recalibration (paired one-tailed t-tests). Diagrams show mean values ± standard errors calculated across subjects. All reported P-values are Bonferroni-corrected for multiple comparisons within each measure (*** P<.001, ** P<.01, n.s. P≥.10). Positive angles denote counterclockwise rotations.

After each movement, subjects visually estimated their pointing direction by means of a trackball-guided cursor (Figure 2B). For each trial, the difference between the *estimated* pointing direction (EPD) and the *motor* pointing direction (MPD) – which we will refer to as the *perceived pointing direction* (PPD = EPD – MPD) – provided us with a relative measure of subjects’ perception of their movements. Importantly, the perceived pointing direction captured any component of the feedback manipulation and thus of the visual prediction error which subjects attributed to internal causes. By way of illustration, the perceived pointing direction should match the feedback manipulation if subjects entirely attributed the prediction error to internal causes (red line in Figure 3A). Vice versa, subjects’ perceived pointing direction should equal 0° (i.e. the estimated and the motor pointing direction should be identical) if subjects entirely attributed the prediction error to external causes (green line in Figure 3A).

The *internally attributed component* of the prediction error should – among other factors – depend on the *absolute error size*. Specifically, the results of earlier studies suggest that subjects should perceive large prediction errors less likely as internally caused than small prediction errors [13], [14], [15], [16], [17]. Accordingly, the share of the internally attributed component in relation to the absolute error size should decrease if the absolute error size increases. If true, this would allow us to gradually manipulate subjects’ causal attribution of prediction errors.

Indeed, when plotting the perceived pointing direction versus the manipulation applied to the visual feedback (blue curve in Figure 3A), we obtained an s-shaped curve, with clockwise manipulations changing the perceived pointing direction in a clockwise manner and, vice versa, counterclockwise rotations inducing a counterclockwise change. For manipulations as small as ±5° and ±10°, subjects’ perceived pointing direction strongly reflected the feedback manipulation (red line in Figure 3A). In contrast, if manipulations were as large as ±20° and ±40°, the estimated pointing direction resembled rather the motor pointing direction (green line in Figure 3A) than the visual feedback direction. This indicated a subproportional relationship between large prediction errors and the internally attributed error component.

To quantify the *internally attributed share* of the prediction error, we defined the *relative weight of visual information* in subjects’ perceptual estimates [18], namely by dividing subjects’ offset-corrected perceived pointing direction by the manipulation applied to the visual feedback in the same trial (Figure 3B, also compare Methods and Figure S1A). This quotient equals 1 if and only if the perceived pointing direction matches the angle of feedback rotation (red line in Figure 3A), which would correspond to an entirely internal attribution of the prediction error. The quotient equals 0 if and only if the estimated pointing direction and the motor pointing direction are identical (green line in Figure 3A), which would correspond to an entirely external attribution of the prediction error.

As expected, the relative weight of visual information – representing the internally attributed share of the prediction error – decreased with increasing prediction error size. Specifically, for manipulations of ±5° and ±10°, the relative weight of visual information was 0.54 and 0.57, respectively. This means that if the prediction error was small, subjects attributed comparable shares of this error to internal and external causes. However, for manipulations of ±20° and ±40°, the visual weight decreased to 0.38 and 0.19, respectively. Here, subjects rather attributed the prediction error to external than to internal causes.

In order to analyse the influence of feedback manipulations on the relative weight of visual information statistically, we conducted a repeated-measures ANOVA with the factors *orientation* (counterclockwise, clockwise) and *amount* of manipulation (5°, 10°, 20°, 40°). We found a significant main effect of amount of manipulation (F(3, 30) = 16.66, P<.001). There were no significant main effect of orientation (F(1, 10) = 1.84, P = .205) and no significant interaction (F(1.26, 12.56) = 0.09, P = .828). We therefore pooled the data across counterclockwise (original data values) and clockwise manipulations (data values multiplied by −1) of the same amount (see Figure 3B).

**Figure 4:**
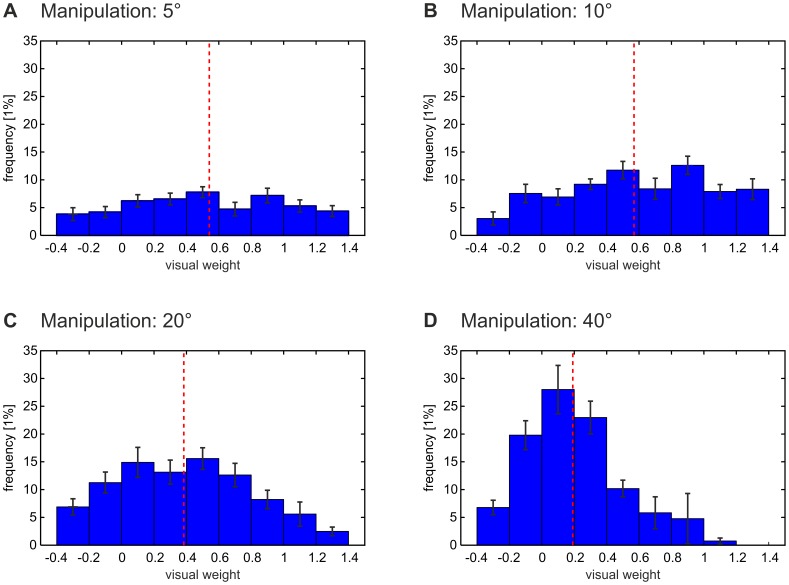
Distribution of the relative weight of visual information. These histograms (mean ± standard error) display the density distribution of the relative weight of visual information across feedback trials. The relative weight of visual information was defined as the quotient of subjects’ offset-corrected perceived pointing direction and the feedback manipulation applied in the same trial (compare Figure 3B). For all amounts of feedback manipulation (5° in A, 10° in B, 20° in C, 40° in D), the histogram exhibits one single peak, indicating a *unimodal* density distribution. This peak corresponds to the mean relative weight of visual information (broken red line). This finding shows that subjects *integrated* the internally predicted and the actual sensory consequences of their actions on the level of *individual* trials when estimating their pointing direction. Alternatively, subjects could have based their estimates solely on internal signals in one trial (corresponding to a relative visual weight of 0) while relying entirely on visual information in another (corresponding to a relative visual weight of 1). This would have resulted in bimodal density distributions, which are not supported by our data. To further support this notion, we statistically examined the distribution of the relative visual weight in feedback trials separately for each subject (n = 11) and for each amount of feedback manipulation (5°, 10°, 20°, 40°). We applied the *Shapiro-Wilk test* to each of these 44 distributions, testing the null hypothesis that the sample came from a normally distributed population. We found that the null hypothesis was tenable in 41 of the 44 samples (P≥.05, uncorrected). This indicates that, indeed, the relative visual weight was normally and therefore unimodally distributed, both across subjects and feedback manipulations.

Additional post-hoc tests showed that the internally attributed share – as measured by the relative weight of visual information – decreased significantly if the amount of visual manipulation increased from 10° via 20° to 40° (P = .007, r = 0.35 and P<.001, r = 0.39 respectively, planned paired one-tailed t-tests to verify the assumption that weight(5°)>weight(10°)>weight(20°)>weight(40°), Bonferroni-corrected for multiple comparisons). However, no significant difference could be found between amounts of manipulation of 5° and 10° (P≥.10). Note that, for all amounts of manipulation, the relative visual weight differed significantly from 0 (one-sample one-tailed t-tests, Bonferroni-corrected for multiple comparisons, see Figure 3B). These findings indicate that the internally attributed share was significant for all prediction errors – despite the fact that these errors were experimentally and, therefore, externally caused – and that the internally attributed share decreased significantly with increasing error size. Note that the internally attributed share of the prediction error across trials showed a density distribution which supports no dichotomous attribution to either internal or, alternatively, external causes, but which rather suggests a continuous attribution mechanism (for details, see Figure 4).

Feedback trials thus allowed us to gradually manipulate the internally attributed component of visual prediction errors – which, according to our hypothesis, should explain the recalibration of subjects’ internal predictions about the visual consequences of their actions.

### Recalibration of Internal Sensory Predictions

To study the recalibration of internal sensory predictions on a *trial-by-trial* basis [19], [20], we used *perceptual probe trials* (Figure 2C), which we presented in alternation with the feedback trials. Perceptual probe trials were identical to feedback trials apart from the fact that subjects received *no* visual feedback about their pointing movements (MPD). When estimating their pointing direction (EPD), subjects consequently needed to rely entirely on internal action-related information. Thus, we assumed that, in perceptual probe trials, the estimated pointing direction and, hence, also the *perceived pointing direction* (PPD = EPD – MPD) reflected an *internal sensory prediction*.

We expected subjects’ internal sensory predictions to recalibrate in response to the prediction errors which we generated in feedback trials. Specifically, this recalibration should be determined by the component of the prediction error which subjects attributed to internal causes. Accordingly, the perceived pointing direction in perceptual probe trials should shift in the orientation of the feedback manipulation applied in the preceding feedback trial. Moreover, the perceived pointing direction in perceptual probe trials should quantitatively mirror the internally attributed component of this feedback manipulation, i.e. the perceived pointing direction in the preceding feedback trial.

When plotting the perceived pointing direction in perceptual probe trials versus the visual manipulation applied in the respective preceding feedback trials (Figure 3C), we obtained another s-shaped curve: counterclockwise manipulations shifted subjects’ estimates of their pointing direction in a counterclockwise fashion and, vice versa, clockwise manipulations induced a clockwise shift – if the manipulations were larger than 5°. This suggests that prediction errors resulted in a recalibration of subjects’ internal sensory predictions, even if these prediction errors were externally caused. The shape of the plot also suggests that the recalibration of subjects’ internal sensory predictions related to the absolute error in a less than proportional manner – thus resembling the internally attributed component of the prediction error (compare Figure 3C to Figure 3A).

To quantitatively compare how visual prediction errors of different size recalibrated subjects’ internal sensory predictions, we defined the *relative recalibration* by dividing the offset-corrected perceived pointing direction in perceptual probe trials by the manipulation applied in the preceding feedback trial (Figure 3D, compare Methods and Figure S1B). This quotient equals 1 if and only if a subject’s perceived pointing direction in a perceptual probe trial matches the visual feedback rotation in the preceding feedback trial (red line in Figure 3C). The quotient equals 0 if and only if the estimated pointing direction and the motor pointing direction are identical (green line in Figure 3C).

If the recalibration of internal sensory predictions was proportional to the absolute prediction error, the relative recalibration would be constant across feedback manipulations. In contrast, if the recalibration of internal sensory predictions reflected the attribution of prediction errors to internal causes, then the relative recalibration should – along with the relative weight of visual information – decrease for increasing amounts of feedback manipulation. Indeed, the relative recalibration showed a maximum of 0.20 at rotations of ±10° and decreased via 0.14 at ±20° to 0.07 at ±40° of rotation (Figure 3D).

To investigate the relative recalibration statistically, we performed a repeated-measures ANOVA with the factors *orientation* (counterclockwise, clockwise) and *amount* of manipulation (5°, 10°, 20°, 40°). We found a significant main effect of amount of manipulation (F(1.77, 17.65) = 4.81, P = .025). There were no significant main effect of orientation (F(1, 10) = 1.29, P = .283) and no significant interaction (F(1.47, 14.69) = 1.18, P = .319). We therefore pooled the data across counterclockwise and clockwise manipulations of the same amount (see Figure 3D).

The relative recalibration differed significantly from 0 for all amounts of manipulation except for those of 5° (one-sample one-tailed t-tests, Bonferroni-corrected for multiple comparisons, Figure 3D). Apparently, prediction errors needed to exceed a certain *threshold* to recalibrate subjects’ internal sensory predictions. However, when exceeding this threshold, one *single* error was sufficient to induce recalibration. The significant main effect of amount of manipulation was explained by post-hoc tests which showed that the relative recalibration decreased significantly if the visual manipulation increased from 10° via 20° to 40° (P = .041, r = 0.18 and P = .015, r = 0.41 respectively, planned paired one-tailed t-tests to verify the assumption that recalibration(5°)>recalibration(10°)>recalibration(20°)>recalibration(40°), Bonferroni-corrected for multiple comparisons, Figure 3D). These findings show that – in quantitative terms – the recalibration of internal sensory predictions could not be explained by the absolute size of the prediction error alone.

**Figure 5:**
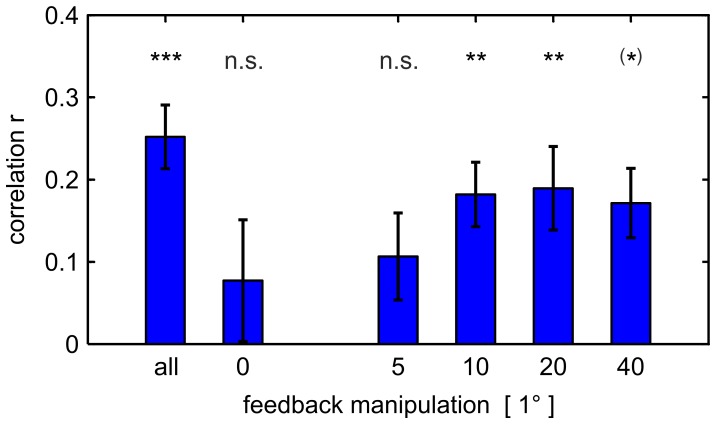
Trial-by-trial recalibration of internal sensory predictions. To investigate the recalibration of internal sensory predictions on a trial-by-trial basis, we performed a linear regression analysis using the perceived pointing direction in feedback trials (Figure 3A) to predict the perceived pointing direction in the consecutive perceptual probe trials (Figure 3C). This analysis was applied to *single* manipulation values as well as to *all* manipulation values. When analysing the correlation coefficients obtained for single manipulation values by a repeated-measures ANOVA, we found no significant main effect of orientation of manipulation (F(1, 10) = 0.66, P = .435), no significant main effect of amount of manipulation (F(3, 30) = 0.70, P = .557) and no significant interaction (F(3, 30) = 2.35, P = .092). We therefore pooled the correlation coefficients across counterclockwise and clockwise manipulations of the same amount. The figure displays the correlation coefficient *r* for *all* manipulation values, for *veridical* feedback (i.e. manipulations of 0°) and for *single* amounts of manipulation of 5°, 10°, 20° and 40° (mean ± standard error across subjects, compare Table S1 for details). If the amount of visual feedback manipulation was larger than 5°, the internally attributed component of the visual prediction error in a given feedback trial explained the recalibration of subjects’ internal sensory predictions. All reported P-values are Bonferroni-corrected for multiple comparisons (one-sample one-tailed t-tests, *** P<.001, ** P<.01, (*) P<.10, n.s. P≥.10).

Instead, the recalibration of internal sensory predictions in a given trial should – according to our hypothesis – be explained by the internally attributed component of the prediction error in the preceding trial. To test this idea directly, we ran a linear regression analysis using the perceived pointing direction in individual feedback trials to predict the perceived pointing direction in the consecutive perceptual probe trials (for details, see Table S1). For ten of our eleven subjects, this linear model was significant. On average, the correlation coefficient within each subject was 0.252±0.039 (mean ± standard error), indicating a positive correlation of moderate effect size (Figure 5). The average regression coefficient was 0.219±0.037, i.e. an increase of the perceived pointing direction in a feedback trial by 10° would, on average, have resulted in an increase of the perceived pointing direction in the following perceptual probe trial by approximately 2°. To minimise the effect of noise inherent to the estimation of the perceived pointing direction, we tested the correlation not only on the level of individual trials, but also across average perceived pointing directions (feedback trials versus perceptual probe trials, matched by visual manipulation). In the latter case, we found a correlation coefficient of 0.56±0.09 (mean ± standard error) within each subject, which emphasised the importance of causal error attribution in recalibration.

The correlation between the error component which subjects attributed to internal causes and the consecutive recalibration of the perceived pointing direction – which we calculated across errors of *varying* size – could have possibly been confounded by the absolute *size* of the errors. Specifically, the correlation might have resulted from a decrease of the relative recalibration for increasing absolute error size which was independent of any causal attribution of feedback errors. For instance, such decrease could have been caused by reduced sensitivity of recalibration to large errors [13].

Therefore, we repeated the trial-by-trial regression analysis separately for each manipulation angle (Figure 5, calculated on the level of individual trials). These analyses again revealed positive correlations irrespective of whether the analysed perceptual probe trials were preceded by visual manipulations of either 10° or 20° or 40°, respectively. These correlations suggest that the causal attribution of the prediction error explains the recalibration of subjects’ internal sensory predictions independently of a given absolute error size. In contrast, for both those trials following on veridical feedback (manipulations of 0°) and those trials following on manipulations of 5°, the average correlation coefficient was not significantly different from 0 (one-sample one-tailed t-tests, Bonferroni-corrected for multiple comparisons, compare Figure 5). This finding suggests that, for small visual manipulations, the perceived pointing direction in feedback trials and the perceived pointing direction in the consecutive perceptual probe trials were independent. This is consistent with the fact that small prediction errors resulted in a strong weighting of visual information in subjects’ perceptual estimates (Figure 3B) but did not significantly recalibrate their internal sensory predictions (Figure 3D).

To assess the evidence our data provide in favour of the hypothesis that the causal attribution of error signals guides the recalibration of sensory predictions, we calculated Bayes factors. A Bayes factor quantifies the evidence for a hypothesis relative to the evidence for an alternative hypothesis. In our case, the alternative hypothesis assumed that recalibration is independent of any causal attribution of error signals. We expected the correlation between the error component attributed to internal causes and the consecutive recalibration to be positive (i.e. within the range from 0 to 1), with small correlation coefficients being more likely than large correlation coefficients. Hence, we modelled the prediction of our hypothesis as a half-normal distribution with a mode of 0 and a standard deviation of 0.5, as suggested by Dienes, 2011 [21]. For each absolute feedback manipulation, we calculated a Bayes factor B according to the procedure described by Dienes [21]. Bayes factors of more than 1 provide evidence for the hypothesis over the alternative hypothesis whereas factors of less than 1, vice versa, favour the alternative. Bayes factors above 3 and below 1/3, respectively, can be considered as substantial evidence [22]. Specifically, our Bayes factors allowed us to evaluate the empirical support for the notion that the causal attribution of error signals guides the recalibration of sensory predictions versus the alternative hypothesis that recalibration is independent of any causal attribution of error signals. We found the following Bayes factors: B(10°) = 151.81, B(20°) = 74.57 and B(40°) = 4.62. This is substantial support for the correlation of error attribution and recalibration. For errors of 5°, the Bayes factor was B(5°) = 0.89 and thus did not allow to decide for any of the two hypotheses with certainty. Importantly, the Bayes factor for the correlation between the perceived pointing direction in veridical feedback trials and the perceived pointing direction in the consecutive perceptual probe trials was B(0°) = 0.42. This yielded further evidence in favour of the assumption that, for veridical feedback trials and their consecutive perceptual probe trials, subjects’ perceived pointing directions were independent. The absence of correlation for those trials following on veridical feedback suggests that the overall correlation between error attribution and recalibration, which we found for absolute amounts of error of 10°, 20° and 40°, was unlikely mediated by an unspecific transfer from feedback trials to the consecutive perceptual probe trials, e.g. due to a drift of the perceived pointing direction during the course of the experiment.

Given the independence of both subjects’ *motor* pointing directions (one-sample two-tailed t-test, t(10) = −0.80, P = .444) and subjects’ *estimated* pointing directions (one-sample two-tailed t-test, t(10) = −0.42, P = .685) across feedback trials and consecutive perceptual probe trials, respectively, the systematic change of the *perceived* pointing direction in perceptual probe trials was unlikely mediated by some unspecific behavioural or perceptual bias unrelated to subjects’ actual movements. For detailed analyses and assessments of these biases, please also refer to Discussion S1. Finally, we asked whether any systematic *direction-dependent* differences in subjects’ perceived pointing direction could have interfered with the recalibration of subjects’ internal sensory predictions. In this case, the *motor* pointing direction *per se* should (partially) explain the *perceived* pointing direction (i.e. the difference between the estimated direction and the motor pointing direction). However, in the perceptual probe trials following on veridical feedback trials, the perceived pointing direction did not covary significantly with the motor pointing direction. Within subjects, a correlation coefficient of 0.038±0.0681 (mean ± standard error) was found (one-sample two-tailed t-test, t(10) = 0.56, P = .590). Hence, systematic direction-dependent differences in subjects’ perceived pointing direction which might have possibly interfered with the recalibration of subjects’ internal sensory predictions by the feedback manipulation could not be detected. For the density distribution of the motor pointing direction in space, compare Figure S2. In summary, these findings support – though indirectly – the notion that subjects’ perceived pointing direction in perceptual probe trials indeed captured adaptive changes of internal *movement-related* reference signals, i.e. a recalibration of internal sensory predictions.

### Generalisation of Learning to Motor Behaviour

**Figure 6:**
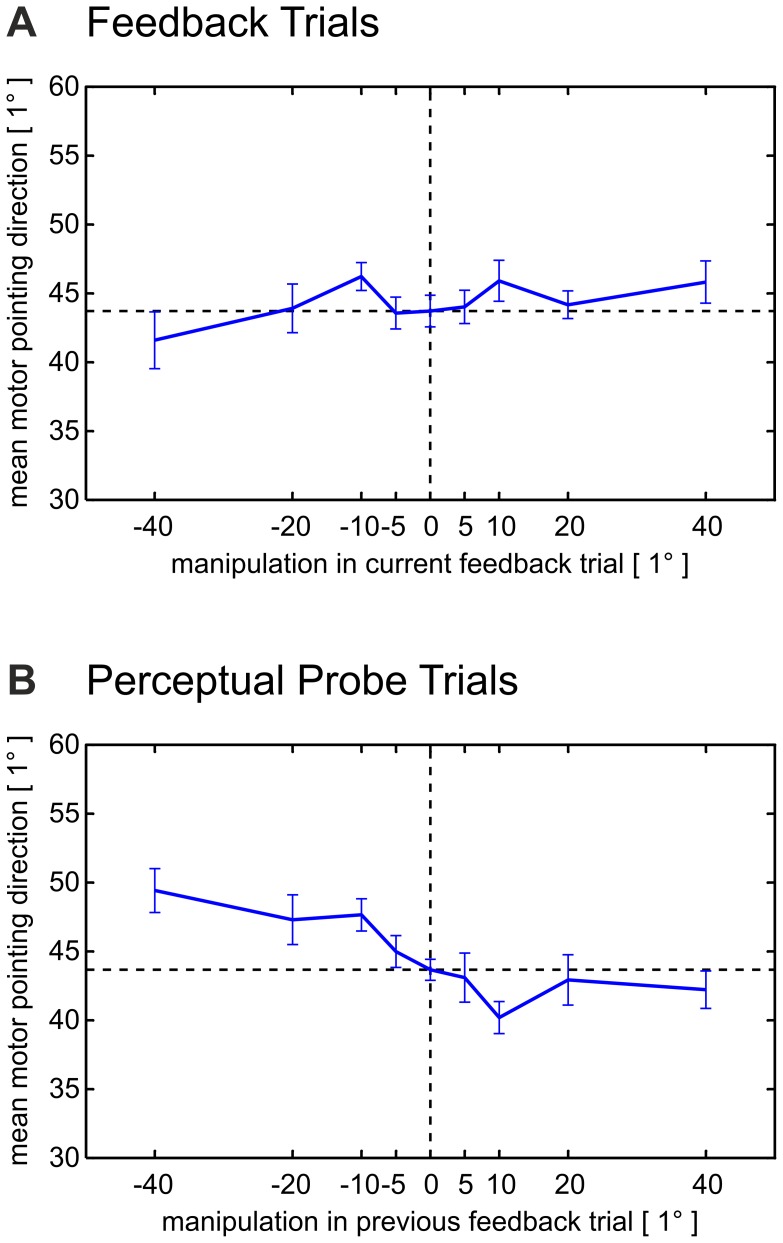
Motor pointing direction as a function of feedback manipulation. *(A) Feedback trials.* The mean *motor* pointing direction in feedback trials (mean ± standard error) is plotted as a function of the visual manipulation, with 0° representing the rightward direction and 90° representing the anterior direction. Subjects showed no systematic online correction of their movement trajectories when presented with deviating visual feedback. *(B) Perceptual probe trials.* The mean *motor* pointing direction in perceptual probe trials (mean ± standard error) is plotted as a function of the visual manipulation applied in the *preceding* feedback trials. Subjects adjusted their motor performance in a manner which compensated for the recalibrated visual movement consequences (compare Figure 3C). Positive angles denote counterclockwise rotations. For the density distribution of the motor pointing direction in space, compare Figure S2.

Based on subjects’ perceived pointing direction in perceptual probe trials, our findings suggest that the manipulation of the visual feedback about subjects’ actions in the preceding feedback trials resulted in a recalibration of subjects’ internal sensory predictions. In addition to the change in subjects’ action perception, one would expect this recalibration of internal sensory predictions to translate into a modification of subjects’ motor performance [9], [23], [24], [25]. Specifically, in perceptual probe trials, subjects should change their motor pointing directions in a manner which compensates for the altered visual consequences of their pointing movements. We provide evidence for this notion by analysing the average *motor* pointing direction in perceptual probe trials as a function of the manipulation applied in the *preceding* feedback trials (Figure 6B).

In comparison to those perceptual probe trials which followed on feedback trials with veridical feedback (0° manipulation), subjects performed movements which on average exhibited a more clockwise direction if the visual feedback had been rotated counterclockwise in the preceding feedback trial. Analogously, if the visual feedback had been rotated in a clockwise fashion, the next movement on average showed a more counterclockwise direction than the movements in the perceptual probe trials which were preceded by veridical feedback. Indeed, feedback manipulation significantly modulated the direction of the movement in the immediately following perceptual probe trial (repeated-measures ANOVA, F(8, 80) = 3.79, P = .001). Moreover, the average *motor* pointing direction in perceptual probe trials (compare Figure 6B) correlated negatively with the average *perceived* pointing direction (compare Figure 3C) in the perceptual probe trials preceded by the same manipulation: analysed on the population level, this correlation exhibited a strong effect size and was highly significant (r(7) = −0.88, P = .002). Subjects thus adjusted their motor performance to the altered visual consequences of the preceding movement. In feedback trials themselves, however, we found no systematic online correction of subjects’ movement trajectories in response to deviating feedback. Here, subjects’ motor pointing direction did not differ significantly across manipulations (repeated-measures ANOVA, F(3.84, 38.42) = 0.94, P = .448, compare Figure 6A).

## Discussion

This study shows that the recalibration of internal predictions about the visual consequences of one’s actions depends on the attribution of visual prediction errors to internal causes. In a virtual-reality setup, we dissociated subjects’ pointing movements and their visual consequences by online rotation of the visual feedback. We thus generated unpredictable discrepancies between the actual and the internally predicted visual consequences of subjects’ actions, i.e. visual prediction errors. Subjects’ visual estimate of their pointing direction allowed us to indirectly quantify the component of the prediction errors which they attributed to internal causes. This internally attributed error component explained the recalibration of subjects’ internal sensory predictions on a trial-by-trial basis: recalibration was not proportional to absolute error size, but instead correlated with the internally attributed error component. We also revealed adaptation in subjects’ motor performance which reflected their recalibrated sensory predictions. More generally, our findings suggest that sensorimotor recalibration depends on a causal interpretation of error information.

### Causal Attribution of Sensory Prediction Errors

We used feedback trials to generate visual prediction errors and to estimate the component of these errors which subjects attributed to internal causes (compare Figure 2B). We generated the prediction errors by rotating the visual feedback about subjects’ movements by various, randomly chosen angles. Subjects were thus faced with an unpredictable discrepancy between the actual and the internally predicted visual consequences of their actions. When estimating their pointing direction, subjects could have attributed this discrepancy – i.e. the prediction error – either to internal causes, or to external causes, or to both internal and external causes. Subjects’ perceived pointing direction reflected both the visual feedback and the motor pointing direction, indicating that subjects attributed the prediction error to internal *and* external causes.

Moreover, the relative weight of visual information [18], which captured the internally attributed error share, showed a unimodal distribution across trials (for details, compare Figure 4), i.e. the values of the relative visual weight scattered around their mean for each amount of manipulation. This distribution does not support a dichotomous error attribution [14], [15] to either internal or, alternatively, external causes, but rather suggests a continuous attribution mechanism on the level of *single* trials. Such partial attribution of prediction errors to internal causes would be consistent with the notion that the perception of one’s actions builds on the *integration* of internal and external action-related cues [26], [27], [28], [29].

Quantitatively, we manipulated the relative weight of visual information in this integration – and thus the internally attributed *share* of the prediction error – by varying the size of the prediction errors: the relative visual weight decreased significantly with increasing amounts of feedback manipulation (see Figure 3B), hereby reflecting the decrease in the probability that the prediction error resulted from internal causes [13], [14], [15], [16], [17]. If the discrepancy between the actual and the internally predicted visual feedback was small, the perceived pointing direction strongly reflected the feedback manipulation (red line in Figure 3A), which indicated that subjects attributed the prediction error mainly to internal causes. In contrast, if the prediction error was large, subjects’ estimated pointing direction rather resembled the motor pointing direction (green line in Figure 3A), which indicated that they attributed the prediction error mainly to external causes. This decrease of the internally attributed error share with increasing error size is consistent with the detection thresholds for visuomotor rotations which were found in comparable tasks [30], [31], [32]: feedback rotations of approximately 13° are commonly detected as externally caused in every second trial. In our study, the internally attributed share was more than 0.5 for prediction errors below this detection threshold and decreased to less than 0.5 for prediction errors above the detection threshold.

### Recalibration of Internal Sensory Predictions through Causal Error Attribution

We analysed the effect of feedback manipulations on subjects' internal predictions about the visual consequences of their actions on the basis of subjects’ perceived pointing directions in perceptual probe trials (compare Figure 2C). Since, in these trials, subjects received no visual feedback about their pointing movements and since these movements were internally guided, i.e. not directed by any external visual goal, subjects’ perceptual estimate of their pointing direction needed to rely entirely on internal cues related to the actual movement. Such cues could be efference copies of motor commands [4], [33], corollary discharge [5], [34] and/or proprioception [35], as discussed in detail elsewhere [8]. Since subjects needed to base the perceptual estimate of their movements entirely on internal information, we assumed that the perceived pointing direction in perceptual probe trials captured a prediction of the visual action consequences which is based on internal information – i.e. it captured an *internal* sensory prediction [6], [36].

Subjects’ internal sensory predictions recalibrated in response to the prediction errors [12] which we generated in feedback trials. Recalibration occurred although these prediction errors were unpredictable. Thus, no consistent discrepancy between the actual and the internally predicted visual action consequences [8], [37] was necessary to induce recalibration. Remarkably, large prediction errors, i.e. amounts of feedback manipulation of 20° and 40°, resulted in a less than proportional recalibration of subjects’ internal sensory predictions in comparison to the recalibration induced by small errors, i.e. amounts of manipulation of 10°. Correspondingly, the relative recalibration, i.e. the quotient of recalibration and preceding feedback manipulation, decreased significantly with increasing error size (Figure 3D). A model which assumes that the recalibration of subjects’ internal sensory predictions increases proportionally with the prediction error thus cannot account for our results because one would then expect the relative recalibration to be constant across feedback manipulations [38], [39]. Instead, the recalibration of subjects’ internal sensory predictions correlated significantly with the internally attributed component of the prediction error, i.e. the perceived pointing direction in the preceding feedback trial (Figure 5 and Table S1). We thus characterised the recalibration of internal sensory predictions employing an indirect measure of each subject’s causal attribution of single prediction errors and following a *trial-by-trial* approach [19], [20]: our results suggest that the recalibration of internal sensory predictions depends on the *attribution* of prediction errors to internal causes. Notably, the correlation between subjects’ perceived pointing direction in feedback trials and their perceived pointing direction in the consecutive perceptual probe trials could also be found *within* errors of equal size (Figure 5). This means that also those differences in the recalibration of subjects’ internal sensory predictions which resulted from errors of the *same* size could be predicted by the component of the prediction error which subjects attributed to their sensorimotor system. In other words, if recalibration occurred, then the causal attribution of the error was decisive for recalibration independently of the given error size.

We found significant recalibration only for those errors which exceeded 5° of amount. The observation that feedback manipulations of 5° failed to induce any recalibration (Figure 3D) despite obtaining significant weight in subjects’ perceived pointing direction (Figure 3B) is suggestive of an *error deadzone*, i.e. errors need to surpass some minimum threshold to recalibrate one’s internal sensory predictions [40], [41]. This absence of adaptation was supported by an absence of correlation between the perceived pointing direction in feedback trials and the perceived pointing direction in the consecutive perceptual probe trials for errors of 5° (Figure 5). The putative error deadzone could reflect an ecologically valuable means to take into account the noise which is inherent to any nervous system, namely by limiting the recalibration of sensory predictions to those errors that exceed the noise level. Actually, the absence of recalibration for errors of 5° parallels the fact that these errors were well below the detection thresholds for visuomotor rotations which were found in comparable paradigms (approximately 13°) [30], [31], [32]. Still, we cannot exclude that our experiment might have failed to capture subjects’ recalibration to small errors due to the limited sensitivity of our methods.

### Possible Constraints of Measuring Recalibration

The systematic change of the perceived pointing direction in perceptual probe trials – which we interpreted as recalibration of subjects’ sensory predictions in response to the experimentally generated prediction errors – could have possibly been mediated by unspecific biases in subjects’ behaviour unrelated to any recalibration of subjects’ sensory predictions, which we address here. With regard to such biases, it is notable that the influence of the feedback manipulation in feedback trials on subjects’ perceived pointing direction in the consecutive perceptual probe trials was unlikely due to an unspecific transfer from feedback trials to perceptual probe trials since, in perceptual probe trials, subjects were not required to reproduce the motor pointing direction of the preceding feedback trial, but were instructed – as for feedback trials – to freely choose any directions in the sector between the subjective directions of anterior and rightwards for their movements. Indeed, subjects did not systematically reproduce the motor pointing direction of feedback trials in the consecutive perceptual probe trials (compare Discussion S1). Analogously, subjects did not systematically reproduce the perceptual estimate of their pointing direction in feedback trials in the consecutive perceptual probe trials (as quantified by the estimated pointing direction, not the perceived pointing direction, compare Discussion S1). Furthermore, the perceived pointing direction, which is a relative measure of subjects’ perception of their actions, was unbiased by the motor pointing direction (compare Results: Recalibration of internal sensory predictions). Likewise, there were no significant differences in gaze direction across feedback manipulations (Figure S3), neither in feedback nor in perceptual probe trials, which could have possibly confounded the influence of the feedback manipulation on subjects’ perception of their pointing direction [42]. Moreover, the change of subjects’ perceived pointing direction in perceptual probe trials was unlikely caused by subjects’ visual reference system being modified by the preceding feedback rotations. In a comparable task, perceptual and motor adaptation to a constant visuomotor rotation were not accompanied by any bias in subjects’ visual estimates of the rightward or the anterior direction [8]. In summary, it thus seems likely that the systematic change of subjects’ perceived pointing direction in perceptual probe trials truly captured a recalibration of subjects’ internal sensory predictions in response to the experimentally generated prediction errors.

### Plastic Distinction of Self- and Externally Caused Sensory Afference

To distinguish between *externally* and *internally caused* sensory afference, sensory predictions about one’s own movements remain to appear suitable internal reference signals [1], [2], [3]. As our findings suggest, these sensory predictions are no absolute references, but rather represent plastic and relative quantities: sensory predictions are internal reference signals which allow a causal attribution of sensory afference *and* in turn are recalibrated themselves based on the causal attribution of the sensory afference. However, given that sensory predictions were thought to capture the part of sensory afference which is *internally* caused [2], [3], how can the prediction error – which supposedly reflects the *externally* caused component of the sensory afference – then be (partially) attributed to *internal* causes? We propose that this inference builds on additional information associated with the prediction errors. Such disambiguating information could result from sensorimotor processes directly [43], [44], for instance in the form of error size [13], [16], error systematics [45] and accompanying sensory events in other modalities [27]. The causal attribution of error signals could also be informed by one’s prior assumptions about the world and oneself [46], [47], by one’s goals [48] and by the reward [49] or the affective outcomes [50] connected with one’s actions. The role of *implicit* and *explicit* processes in this attribution thereby remains a question for future research [51], [52]. In contrast to the aforementioned sources of information, the particular ecological value of internal sensory predictions consists – despite the continuous need for recalibration – in their rapid and reliable availability [27], [53].

### Generalisation of Learning to Motor Behaviour

Internal sensory predictions have been shown to subserve both *perception* [11], [54] and *control* of one’s actions [8], [9], [23]. Indeed, subjects’ average motor pointing direction in perceptual probe trials changed in a manner which compensated for the predicted change in the visual consequences of the pointing movements (compare Figure 6B, also see [24], [25]). Specifically, in perceptual probe trials, subjects’ average *motor* pointing direction and their average *perceived* pointing direction correlated significantly in a negative manner (compare Results: Generalisation to motor behaviour). This finding provides, to our knowledge, first evidence for previous assumptions made in research of the *motor* domain which suggested that causal inferences about motor errors determine the degree of motor adaptation [16]: Wei and colleagues [16] proposed that the *relevance* of motor errors, i.e. the probability that a motor error results from intrinsic causes, would explain the subproportional relation between the size of movement errors and adaptive changes in motor behaviour. This relation has been frequently observed, e.g. in the adaptation of goal-directed reaching to visual movement errors [16], in saccadic adaptation to visual saccade errors [55] or in the adaptation of straight movements in disturbing force fields [56]. Our findings specify this notion by demonstrating that the relevant errors are those errors which are attributed to internal causes. Our study thus provides evidence for a general role of causal inferences about error information in sensorimotor learning: Sensorimotor recalibration occurs in response to those errors which – from the perspective of the nervous system – likely originate from internal causes. Moreover, our findings suggest that both the recalibration of motor performance and the recalibration of internal sensory predictions build on adaptive changes in a shared internal forward model [9], [23].

The notion of a shared internal sensory prediction is supported by previous visuomotor adaptation experiments [8], [30], [31]. However, motor and sensory recalibration could still be processes which occur simultaneously [24], [25], [57], yet dissociate from each other [49], [58], [59]. Such dissociation might thereby be grounded in the differential objectives of motor and sensory learning: Unlike the recalibration of sensory predictions about one’s movements, motor learning does not primarily aim at establishing congruence between the actual and the internally predicted sensory consequences of one’s movements. Instead, motor learning can be understood as optimising the achievement of external goals [12], [60]. The degree to which prediction errors are relevant to motor learning thus not only depends on the causal attribution of these errors to internal versus external causes [16], [43], but also on (reward) prediction errors related to the achievement of one’s goals [49].

### Causality and Systematics of Errors in Learning

The relevance which error signals receive in sensorimotor learning could also reflect the *systematics* in the occurrence of errors [59]: While our findings emphasise the role of error causality for *unsystematic* errors, the causal attribution of errors to external versus internal sources might be equally important for learning from *systematic* errors [45]. This might be surprising at first glance since – in contrast to the case of unsystematic errors – it seems plausible to compensate for any systematic error irrespective of whether this error is internally or externally caused. Yet, the way *how* one compensates for internally versus externally caused systematic errors might be different: First, the causal attribution of *systematic* errors to an *external* cause could be understood as a change of context. The causal attribution of errors to a specific external context is essential to allow *context-dependent* learning. Only thus can we learn to reliably and efficiently predict the sensory consequences of our movements in specific external contexts (e.g. with versus without wearing glasses). Second, the causal attribution of *systematic* errors to *internal* causes is decisive if internal changes within the sensorimotor system are to induce learning which is independent of external contexts (e.g. transfer of rehabilitation training to everyday tasks). Thus, the causality of errors appears relevant to sensorimotor learning also in view of error systematics.

In summary, the causal attribution of error signals may provide a general framework for understanding the plasticity of both perception and control of one’s actions.

## Materials and Methods

### Subjects

Eleven right-handed healthy subjects (4 women, 7 men, mean age ± standard error: 28.09±1.77 years) with normal or corrected-to-normal visual acuity participated in the study. All subjects gave written informed consent according to the Declaration of Helsinki. The study was approved by the ethics committee of the University of Tübingen, Germany.

### Experimental Setup

Subjects performed pointing movements in a *virtual-reality setup* [8], [30], [31] in which the visual consequences of their movements could be manipulated in real time (compare Figure 2A). They were seated in front of a horizontal board with their heads stabilised in a head-and-chin rest. Via a horizontal mirror, subjects viewed a computer screen which was positioned horizontally and upside-down above the mirror. Since the mirror was located halfway between the board and the screen, this screen – on which visual feedback about subjects’ movements was provided – appeared to be in the plane of the horizontal board. Subjects were instructed to place both hands on the board and therefore could not see their hands. To reduce spatial information for orientation, we conducted the experiment in darkness.

We instructed subjects to perform their pointing movements with their right index finger on the board surface. We recorded the position of the fingertip online using a three-dimensional ultrasound-based motion-tracking system (*Zebris CMS 70 P*, Isny, Germany). Via the mirror-screen setup, we could provide subjects with visual feedback about the position of their right index fingertip in real time (60 Hz).

The experiment was realised using *Cogent Graphics* developed by John Romaya at the Laboratory of Neurobiology at the Wellcome Department of Imaging Neuroscience (London, UK) and the *Psychophysics Toolbox* [61], [62].

### Experimental Procedure

Subjects performed centre-outward-and-back *pointing movements* with their right index finger on the board surface (see Figure 2A). A haptic marker on the board defined the starting and end point of these movements. On the screen, this marker was veridically represented by a white disc (0.25° radius). Subjects were instructed to move out and back as straight and fast as possible. In each trial, the pointing amplitude was indicated by a briefly flashed circle (300 ms duration, 9.0° radius) whose centre corresponded to the starting point. Subjects did not receive specific visual targets for their pointing movements, but were instructed to freely choose any directions in the upper right quarter of the initially flashed circle, i.e. the sector between the subjective directions of anterior and rightwards.

The two *experimental conditions*, feedback trials and perceptual probe trials, were presented alternately. In *feedback trials* (Figure 2B), subjects received online visual feedback about their movement. Specifically, subjects’ fingertip was represented by a white disc (radius: 0.20°) which moved in an experimentally controlled relation to the fingertip. This *visual feedback* was either veridical or, alternatively, rotated around the starting point by various angles. In each trial, the rotation angle was randomly drawn from a discrete uniform distribution over the values 5°, 10°, 20°, 40° (counterclockwise rotation), −5°, −10°, −20°, −40° (clockwise rotation) and 0° (veridical feedback). To prevent online correction of subjects’ movements, we provided visual feedback only for the peripheral part of the movements (amplitude >4.5°) and limited feedback presentation to 1000 ms after movement onset. In *perceptual probe trials* (Figure 2C), subjects did not receive any visual feedback about their movement. Here, we only presented the central white disc and the circle which indicated pointing amplitude.

After each pointing movement, subjects visually estimated the direction of their actual movement by placing – with their left hand – a trackball-guided cursor in the respective direction. The procedure by which subjects estimated their pointing direction was identical in feedback and perceptual probe trials. Note that subjects were not required to reproduce their movement. Instead, rotation of the trackball was transformed into circular movement (4.5° radius) of another white disc (0.15° radius) around the starting position (0.25° radius). When having placed this disc relative to the starting position in the *estimated direction* of their movement, subjects confirmed their estimate by pressing the right trackball button. Note that subjects were instructed to fixate their gaze on the starting point during movement execution, but that fixation was not required during the subsequent estimation of the movement direction.

During the measurement, trials were declared invalid if the amplitude of the executed movement was less than half of the instructed amplitude or if subjects provided no perceptual estimate of the pointing direction. Altogether, each subject needed to complete 180 valid feedback trials and 180 valid perceptual probe trials. To ensure that subjects could execute the task correctly, we had them perform 20 practice trials in advance (first a block of 10 veridical feedback trials, then a block of 10 perceptual probe trials).

### Data Analysis

We recorded each movement trajectory for offline analysis. The direction of the pointing movement was defined as the direction of a straight line which we fitted to the horizontal position samples acquired during the outward movement of the finger by means of a linear regression analysis (for details, compare [8]).

During offline analysis, we discarded those trials in which the curvature of the trajectory was particularly pronounced, thus ensuring to remove those trials in which subjects might have corrected their movement online. Specifically, if the maximum deviation of the outward movement from the straight line connecting the starting point and the end point of the outward movement exceeded 2.25° (i.e. one quarter of the instructed movement amplitude), this trial was excluded. Furthermore, to remove trials with sampling artefacts specific to our ultrasound-based motion-tracking method, we excluded those trials in which the absolute movement velocity exceeded 120°/s. As we evaluated perceptual probe trials in terms of the manipulation applied in the respective preceding feedback trials, we further discarded all perceptual probe trials which immediately followed on invalid or discarded feedback trials.

On average, subjects performed 185.55±3.64 feedback trials and 183.82±3.32 perceptual probe trials (mean ± standard error). After applying the above exclusion criteria, we evaluated 171.09±3.60 feedback trials and 163.45±6.68 perceptual probe trials. The number of evaluated trials did not differ significantly across manipulation angles, neither for feedback trials (repeated-measures ANOVA, F(3.55, 35.47) = 1.32, P = .282) nor for perceptual probe trials (repeated-measures ANOVA, F(3.52, 35.22) = 1.59, P = .205). In all analysed feedback trials, pointing amplitude was more than 4.5°, which confirms that subjects indeed received visual feedback in these trials.

To account for our *within-subject design*, we analysed subjects’ performance using two-way repeated-measures ANOVAs with the factors orientation and amount of manipulation (see Results). We used Mauchly’s test to check if the assumption of sphericity was tenable. Whenever sphericity was violated, we corrected the degrees of freedom according to Greenhouse and Geisser. We performed post-hoc analyses (paired one-tailed t-tests, Bonferroni-corrected for multiple comparisons) only if the according main effect was significant.

In all figures, we provide mean values and standard errors calculated across subjects. Figure 3A and 3C display subjects’ *offset-corrected perceived pointing directions*. *Within subjects*, we offset-corrected the perceived pointing direction in feedback trials (see Figure 3A and Figure S1A) by subtracting the mean perceived pointing direction of feedback trials with veridical feedback. Analogously, we offset-corrected the perceived pointing direction in perceptual probe trials (see Figure 3C and Figure S1B) by subtracting the mean perceived pointing direction of those perceptual probe trials preceded by veridical feedback. We performed such offset-correction to account for systematic differences between the estimated and the motor pointing direction which are commonly revealed in comparable tasks [24], [25], [30], [31].

To account for *between-subject* variance in our figures, further normalisation was performed as suggested by Masson and Loftus [63]: “Normalization is based on the deviation between a subject’s [i] overall mean [m_i_], computed across that subject’s scores in each condition, and the grand mean [GM] for the entire sample of subjects […]. That deviation is subtracted from the subject’s score [X] in each condition [j] (i.e., X_ij_ – (M_i_ – GM)) to yield a normalized score for that subject in each condition […].” Accordingly, across-subject averages remained unchanged by this normalisation while the measures of variability (specifically, the standard errors) excluded the between-subject variability and represented the *average within-subject variability* only.

## Supporting Information

Figure S1 **Perceived pointing direction before offset-correction.** Panel (A) displays the perceived pointing direction (PPD, mean ± standard error) in *feedback trials* as a function of the manipulation applied to the visual feedback. Analogously, Panel (B) shows the PPD in *perceptual probe trials* as a function of the visual manipulation in the *preceding* feedback trial. Unlike in Figures 3A and 3C, the data displayed here are original data before offset-correction: the estimated direction of the pointing movement was systematically shifted in relation to the motor pointing direction, which is a common finding in comparable tasks [8], [24], [25], [30], [31].
(TIF)

Figure S2 **Spatial distribution of the motor pointing direction.** Subjects did not receive specific visual targets for their pointing movements, but were instructed to freely choose any directions in the upper right quarter of the initially flashed circle, i.e. the sector between the subjective directions of anterior (90°) and rightwards (0°). In fact, subjects’ motor pointing directions were mainly distributed between 10° and 70°, both in feedback (A) and perceptual probe trials (B), as the histograms illustrate (mean ± standard error across subjects).
(TIF)

Figure S3 **Gaze direction during movement execution.** Since gaze direction can modify the perception of one’s hand position [42], systematic differences in gaze direction could have possibly confounded the influence of feedback manipulations on subjects’ perceived pointing direction. We therefore required subjects to fixate on the starting point of their pointing movements during movement execution and, additionally, analysed subjects’ gaze direction as a function of feedback manipulation. We recorded the position of subjects’ left eye using a video-based dark-pupil tracking method (ViewPoint Eye Tracker, Arrington Research Inc., Scottsdale, USA). Eye position was sampled at 50 Hz and processed offline. After filtering the data (second-order 10 Hz Chebyshev digital low-pass filter Type II, R = 3), we removed artefacts owing to eye blinks by means of an eye position criterion. We analysed subjects’ eye position during a period of 1000 ms starting at the moment that the finger-centre distance exceeded 4.5° visual angle. In other words, the epoch during which gaze was evaluated equalled the maximum possible period of feedback presentation during feedback trials (see Methods, Experimental Procedure). For each trial, we determined the mean position of gaze on the movement plane. We then calculated the direction of this position from the starting point of the pointing movements and, finally, the direction of gaze *relative* to the direction of subjects’ pointing movement. Eye movements were measured and evaluated for ten of our eleven subjects. Panel (A) shows subjects’ *relative gaze direction* as a function of the visual manipulation in *feedback trials* (mean ± standard error). Panel (B) shows the relative gaze direction in *perceptual probe trials* as a function of the preceding feedback manipulation. Feedback manipulation did not modify the relative gaze direction significantly, neither in feedback trials (repeated-measures ANOVA, F(8, 72) = 1.26, P = .279) nor in perceptual probe trials (repeated-measures ANOVA, F(8, 72) = 0.46, P = .882). Thus, the differences in subjects’ perceived pointing direction across feedback manipulations cannot be explained by systematic differences in gaze direction.
(TIF)

Table S1 **Trial-by-trial recalibration of internal sensory predictions.** A linear regression analysis revealed that subjects’ internal predictions about the sensory consequences of their actions were recalibrated on a trial-by-trial basis. Specifically, we performed a linear regression analysis which used the perceived pointing direction in feedback trials to predict the perceived pointing direction in the consecutive perceptual probe trials. For each subject, the table reports the degrees of freedom (df), the correlation coefficient (r), the P-value (P) and the regression coefficient (m) obtained in this analysis.
(PDF)

Discussion S1 **Possible constraints of sensorimotor recalibration.**
(PDF)
