# Supplementary figures and images for: Sensorimotor Recalibration Depends on Attribution of Sensory Prediction Errors to Internal Causes
Source: PLoS One. 2013 Jan 24;8(1):e54925. doi: 10.1371/journal.pone.0054925 (PMC3554678; doi:10.1371/journal.pone.0054925)

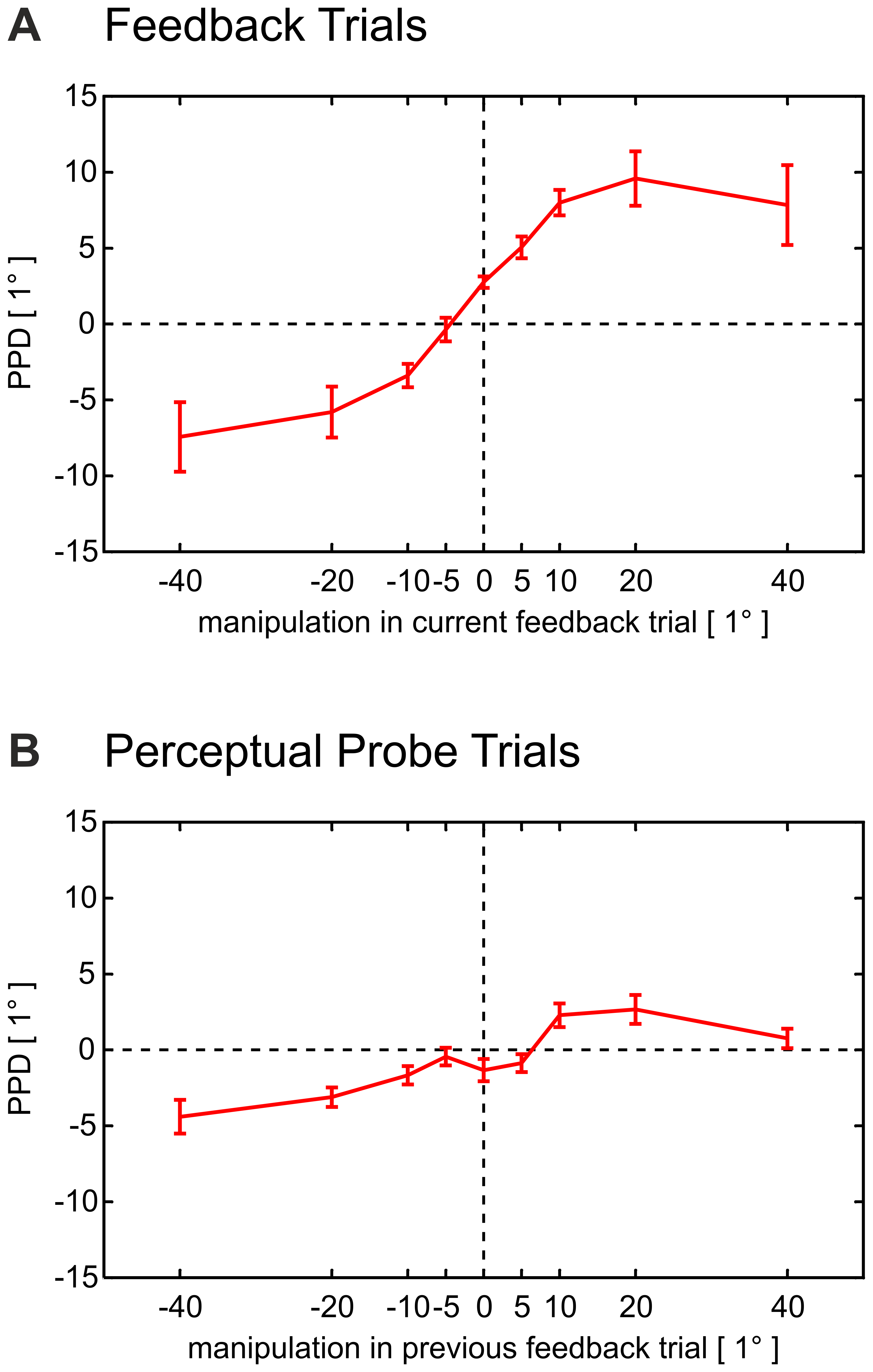

Supplement: Figure S1 — Perceived pointing direction before offset-correction. Panel (A) displays the perceived pointing direction (PPD, mean ± standard error) in feedback trials as a function of the manipulation applied to the visual feedback. Analogously, Panel (B) shows the PPD in perceptual probe trials as a function of the visual manipulation in the preceding feedback trial. Unlike in Figures 3A and 3C, the data displayed here are original data before offset-correction: the estimated direction of the pointing movement was systematically shifted in relation to the motor pointing direction, which is a common finding in comparable tasks [8], [24], [25], [30], [31]. (TIF) [file pone.0054925.s001.tif]

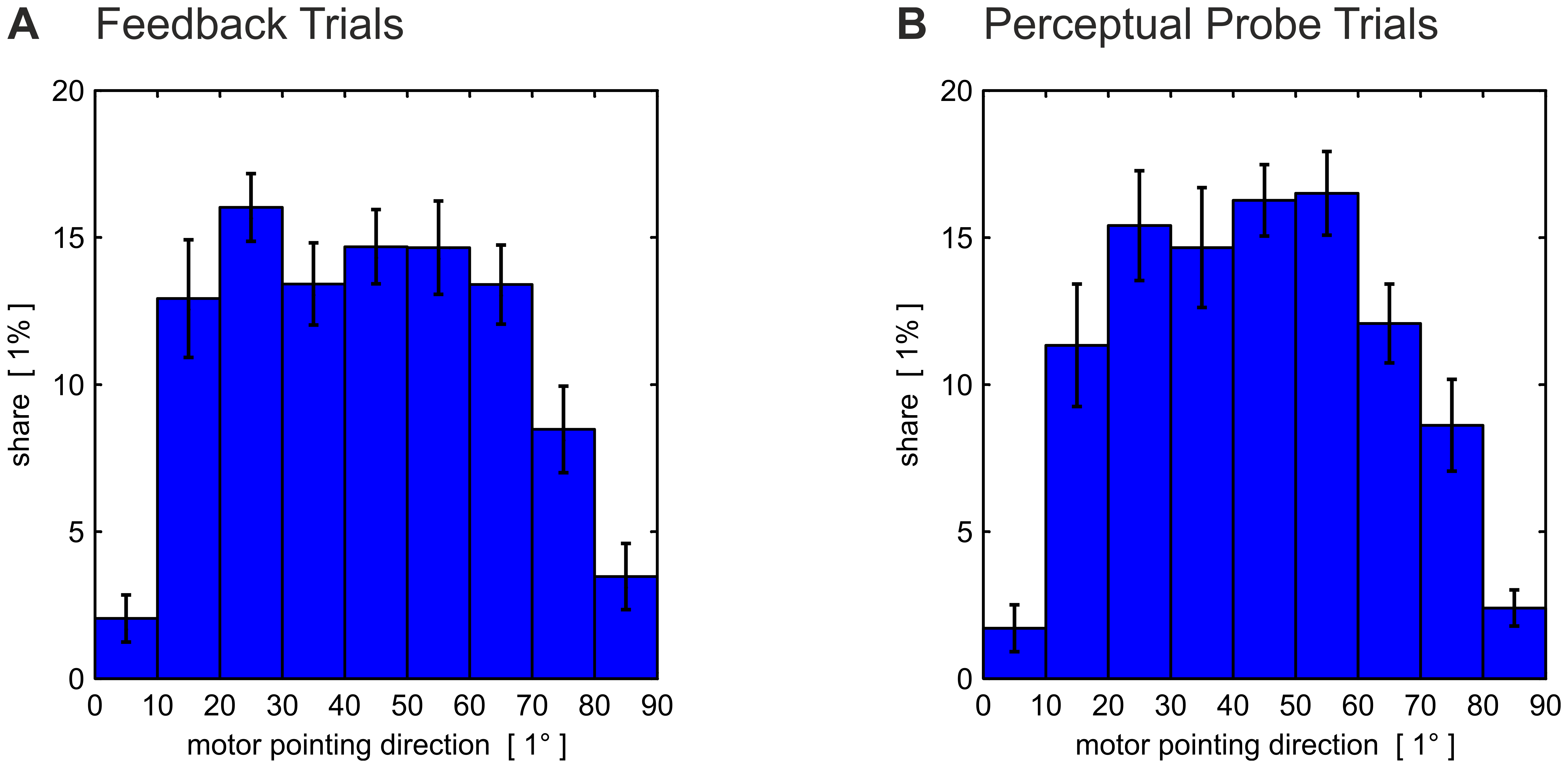

Supplement: Figure S2 — Spatial distribution of the motor pointing direction. Subjects did not receive specific visual targets for their pointing movements, but were instructed to freely choose any directions in the upper right quarter of the initially flashed circle, i.e. the sector between the subjective directions of anterior (90°) and rightwards (0°). In fact, subjects’ motor pointing directions were mainly distributed between 10° and 70°, both in feedback (A) and perceptual probe trials (B), as the histograms illustrate (mean ± standard error across subjects). (TIF) [file pone.0054925.s002.tif]

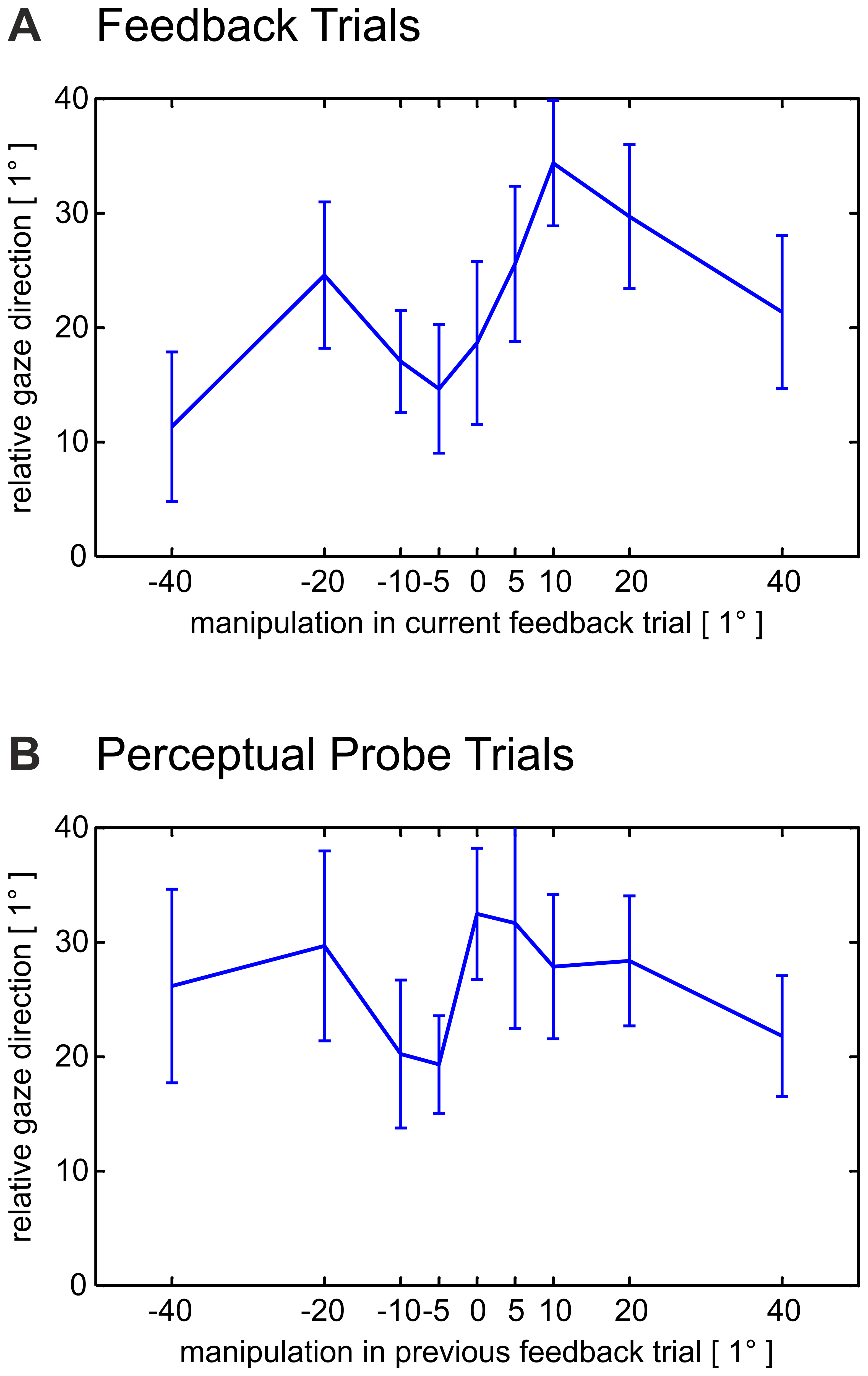

Supplement: Figure S3 — Gaze direction during movement execution. Since gaze direction can modify the perception of one’s hand position [42], systematic differences in gaze direction could have possibly confounded the influence of feedback manipulations on subjects’ perceived pointing direction. We therefore required subjects to fixate on the starting point of their pointing movements during movement execution and, additionally, analysed subjects’ gaze direction as a function of feedback manipulation. We recorded the position of subjects’ left eye using a video-based dark-pupil tracking method (ViewPoint Eye Tracker, Arrington Research Inc., Scottsdale, USA). Eye position was sampled at 50 Hz and processed offline. After filtering the data (second-order 10 Hz Chebyshev digital low-pass filter Type II, R = 3), we removed artefacts owing to eye blinks by means of an eye position criterion. We analysed subjects’ eye position during a period of 1000 ms starting at the moment that the finger-centre distance exceeded 4.5° visual angle. In other words, the epoch during which gaze was evaluated equalled the maximum possible period of feedback presentation during feedback trials (see Methods, Experimental Procedure). For each trial, we determined the mean position of gaze on the movement plane. We then calculated the direction of this position from the starting point of the pointing movements and, finally, the direction of gaze relative to the direction of subjects’ pointing movement. Eye movements were measured and evaluated for ten of our eleven subjects. Panel (A) shows subjects’ relative gaze direction as a function of the visual manipulation in feedback trials (mean ± standard error). Panel (B) shows the relative gaze direction in perceptual probe trials as a function of the preceding feedback manipulation. Feedback manipulation did not modify the relative gaze direction significantly, neither in feedback trials (repeated-measures ANOVA, F(8, 72) = 1.26, P = .279) nor in perceptual probe trial [file pone.0054925.s003.tif]
