# Supplementary material for: Sensorimotor Recalibration Depends on Attribution of Sensory Prediction Errors to Internal Causes
Source: PLoS One. 2013 Jan 24;8(1):e54925. doi: 10.1371/journal.pone.0054925 (PMC3554678; doi:10.1371/journal.pone.0054925)
Supplement: Table S1 — Trial-by-trial recalibration of internal sensory predictions. A linear regression analysis revealed that subjects’ internal predictions about the sensory consequences of their actions were recalibrated on a trial-by-trial basis. Specifically, we performed a linear regression analysis which used the perceived pointing direction in feedback trials to predict the perceived pointing direction in the consecutive perceptual probe trials. For each subject, the table reports the degrees of freedom (df), the correlation coefficient (r), the P-value (P) and the regression coefficient (m) obtained in this analysis. (PDF) [file pone.0054925.s004.pdf]

Table S1. Trial-by-trial recalibration of internal sensory predictions.

subject	df	r	P	m
1	166	0.176	0.022	0.134
2	176	0.331	< 0.001	0.303
3	106	0.065	0.505	0.056
4	158	0.211	0.008	0.228
5	177	0.175	0.019	0.152
6	134	0.292	< 0.001	0.165
7	174	0.221	0.003	0.184
8	173	0.236	0.002	0.216
9	169	0.538	< 0.001	0.508
10	167	0.374	< 0.001	0.330
11	176	0.151	0.045	0.133
