# Supplementary material for: Sensorimotor Recalibration Depends on Attribution of Sensory Prediction Errors to Internal Causes
Source: PLoS One. 2013 Jan 24;8(1):e54925. doi: 10.1371/journal.pone.0054925 (PMC3554678; doi:10.1371/journal.pone.0054925)
Supplement: Discussion S1 — Possible constraints of sensorimotor recalibration. (PDF) [file pone.0054925.s005.pdf]

Discussion S1. Possible constraints of sensorimotor recalibration.

The correlation between the component of the prediction error which subjects attributed to internal causes and the systematic change of subjects' sensory predictions which we observed in perceptual probe trials could have possibly been mediated by unspecific biases in subjects' motor behaviour or their perceptual reports. First, subjects could have reproduced the motor pointing direction of feedback trials in the following perceptual probe trials. Second, the manipulation of the visual feedback could have induced a common spatial bias of subjects' estimated pointing directions in both feedback trials and the consecutive perceptual probe trials. Each of these biases could have underlain the correlation between subjects' perceived pointing direction in feedback trials and the perceived pointing direction in the consecutive perceptual probe trials. Hence, each bias could challenge our interpretation that the causal attribution of prediction errors guided the recalibration of subjects' sensory predictions.

To exclude the possibility of reproduced motor pointing directions, we calculated the correlation of each subject's motor pointing directions in feedback and consecutive perceptual probe trials. Within subjects, this correlation was -0.071 ± 0.090 (mean ± standard error) and not significantly different from 0 (one-sample two-tailed t-test, $t(10) = -0.80$, $P = .444$). Additionally, we found no significant influence of feedback manipulation on this correlation (repeated-measures ANOVA, $F(8, 80) = 0.45$, $P = .889$). Hence, subjects did not systematically reproduce their motor pointing directions, but chose their motor pointing directions in feedback and consecutive perceptual probe trials independently.

To further exclude that the visual feedback had induced a spatial bias in the estimated pointing direction unrelated to the actual movement, we calculated the correlation of subjects' estimated pointing directions between feedback and consecutive perceptual probe trials. Within subjects, this correlation was -0.038 ± 0.091 (mean ± standard error) and not significantly different from 0 (one-sample two-tailed t-test, $t(10) = -0.42$, $P = .685$). Moreover, there was no significant influence of feedback manipulation on this correlation (repeated-measures ANOVA, $F(8, 80) = 0.62$, $P = .763$). We therefore assume that subjects did not systematically reproduce their estimated pointing directions in consecutive feedback and perceptual probe trials. In other words, a spatial bias in the estimation of the pointing directions which is unrelated to the actual motor pointing directions appears unlikely to explain the systematic change of subjects' perceived pointing directions in perceptual probe trials.
